# History, epidemiology and regional diversities of urolithiasis

**DOI:** 10.1007/s00467-008-0960-5

**Published:** 2010-01-01

**Authors:** Michelle López, Bernd Hoppe

**Affiliations:** 1 Department of Nephrology, Hospital de Niños JM de los Ríos, Caracas, Venezuela; 2 Department of Pediatrics, Division of Pediatric Nephrology, University Hospital of Cologne, Cologne, Germany; 3 Division of Pediatric Nephrology, University Children’s Hospital of Cologne, Kerpenerstr. 62, 50924 Cologne, Germany

**Keywords:** Urolithiasis, History, Epidemiology, Risk factors

## Abstract

Archeological findings give profound evidence that humans have suffered from kidney and bladder stones for centuries. Bladder stones were more prevalent during older ages, but kidney stones became more prevalent during the past 100 years, at least in the more developed countries. Also, treatment options and conservative measures, as well as ‘surgical’ interventions have also been known for a long time. Our current preventive measures are definitively comparable to those of our predecessors. Stone removal, first lithotomy for bladder stones, followed by transurethral methods, was definitively painful and had severe side effects. Then, as now, the incidence of urolithiasis in a given population was dependent on the geographic area, racial distribution, socio-economic status and dietary habits. Changes in the latter factors during the past decades have affected the incidence and also the site and chemical composition of calculi, with calcium oxalate stones being now the most prevalent. Major differences in frequency of other constituents, particularly uric acid and struvite, reflect eating habits and infection risk factors specific to certain populations. Extensive epidemiological observations have emphasized the importance of nutritional factors in the pathogenesis of urolithiasis, and specific dietary advice is, nowadays, often the most appropriate for prevention and treatment of urolithiasis.

## Introduction

Although less frequent than adult stone disease, urolithiasis in the pediatric age group plays an important and increasing role, not only in parts of the world with a high incidence of stone disease such as the Near and Far East, but also in the industrialized countries [1]. Pediatric urolithiasis is associated with significant morbidity, particularly since stones tend to recur, and, thus, should not be underestimated. Even though great progress has been achieved during the past few decades in a better understanding of the etiology, pathophysiology, treatment and prevention of urolithiasis, many aspects still remain controversial and also depend on obvious regional diversities of stone disease.

Urolithiasis has become more common in children over the past few decades as a result of rapid variations in habits and increasing affluence. Changing socio-economic conditions have generated changes in the incidence and type of urolithiasis in terms of both the site and the physico-chemical composition of the calculi. Major variations in worldwide occurrence of urolithiasis have been reported, according to geographical areas and historical periods.

This teaching paper focuses on historical knowledge about recurrent stone disease and aims to clarify differences in epidemiologic data in different regions of the world.

## Historical background

Kidney stone disease has been a well-known entity for centuries. This has been markedly established by different archeological findings, as well as by writings about painful stone colic and therapeutic trials for stone removal [2]. In ancient centuries urolithiasis was often a disastrous disease, with a catastrophic outcome all too often leading to the patient’s death.

Examinations of Egyptian mummies have revealed kidney and bladder stone disease. For example, in 1901, the English archeologist E. Smith found a 5,000-year-old bladder stone at the funeral site of El Amrah, Egypt [2, 3]. However, he reported only four cases of urolithiasis in thousands of examined mummies, which led to the conclusion that stone disease must have been less prevalent in ancient Egypt. Regimens for treatment of diseases of the urinary tract, including stones, had already been found in the papyrus Ebers (1500 BC), being the main origin of our knowledge of old traditional Egyptian medicine. Although the ancient Egyptians were famous for their mummifying techniques, they obviously did not know how to remove kidney or bladder stones [2].

Already in ancient Mesopotamia, knowledge of soluble or insoluble (bladder) stones was available. On various stone tablets, recipes for treatment of different diseases, such as the dissolution of soft kidney and bladder stones, were found. Saltpeter and turpentine oil were known to increase urine production, and pulverized egg-shell, mainly from ostrich eggs, with a high content of calcium carbonate, was ingested to bind lithogenic substances [3, 4]. The harder stones were left for surgical treatment. Hence, our typical current treatment options, and even invasive interventions such as urethral catheterization, were already ‘available’ centuries ago [3, 5]. A specific correlation between urinary tract infection and the development of stones was reported in neither ancient Egypt nor Mesopotamia, although dysuria, hematuria and pyuria were observed, and urethral catheterization in patients with urinary retention, e.g. due to gonorrheal urethritis, was performed.

Uroscopy, for example, was also known as a fundamental diagnostic evaluation, as were diseases of the urinary tract such as bladder or kidney calculi, both already mentioned in the Hippocratic collection five centuries BC [3, 5]. Three centuries BC, Ammonius first mentioned that crushing a bladder stone would make its removal much easier, for which he received his nickname “Lithotomus” = Stein-Schneider (stone cutter) [3]. Regardless of his revolutionary thoughts, surgical procedures for stone removal only became popular centuries later.

Detailed medical literature is also known from ancient India. In the sixth century BC, Sushruta, the main physician to the king of India, first reported in his series of volumes, the Sushruta-Samshita, stone removal via the urethra using a splint. Next, he reported on urolithiasis and its complications, such as infection, anuria and uremia, hence a mostly disastrous outcome in patients with stones. Another famous Hindu author, Charaka, reported in his Samshita on instrumental removal of urethral stones. Sushruta later recommended the so-called Steinschnitt (stone cut) for the treatment of bladder stones. Because many patients died under this procedure, it was only allowed after consent had been given by the sovereign, but it was the only possibility for helping those who had been suffering from severe and ongoing colicky pain for a long time. As this procedure needed skillful undertaking, the profession of the traveling Stein-Schneider (stone cutter, people who removed urinary stones) was later born. These new specialists continued to travel throughout Europe until the late eighteenth century.

The knowledge from ancient India possibly reached central Europe via Persia and ancient Greece. Hippocrates had profound knowledge of stone disease and well described the clinical symptoms of bladder stones. One of the most known Stein-Schneider of the Alexandrine epoch, who lived around 280 BC, introduced a lithotripsy procedure for the treatment of bladder stones. However, such stone removal procedures were originally prohibited by the Hippocratic Oath, possibly due to the severe risks of the procedure [6]. Complications observed at this time were high fevers, urinary fistulas, impotence due to the perineal approach, and even death. Working men (surgeons), who were then not bound by the Hippocratic Oath, were hence the only persons able to perform such procedures.

Since the first century BC, ancient Greek medicine had become available in the Roman Empire. One of the most known physicians of this time was Aulus Cornelius Celsus (ca. 25 BC–ca. 50 AD), who tried to include all medical knowledge into an encyclopedia. In this book he reported on the typical (colicky) abdominal pain of patients with urolithiasis, but he also described the ‘Steinschnitt’ procedure with a perineal incision, which was called the Celsic method and was used up until the late eighteenth century [3, 6]. He recommended that the patient take a long walk and a specific diet before the procedure finally took place after a 1-day fast in a warm room. He also reported on complications, mostly resembling the typical signs of urosepsis, but also post-operative bleeds, fistulas, bladder tamponade, etc. Most of the patients so treated were boys aged 9−14 years, a fact that later led to the postulation that kidney disease was most prevalent in this age group. At the end of the Roman Empire the methods of ancient Greek medicine were lost in Europe. The monks, respected as the ‘intellectuals’ of the fifth to twelfth centuries, were the only persons still knowing and performing the ‘old’ medical procedures, and the monasteries were the only places where such procedures were performed.

Within Arabic–Islamic medicine, stone removal procedures were also known and were frequently reported by Rhazes (865–925) and Ibn Sina (Avicenna, 980–1037), but especially from Abulcasis (936–1013 AD), who worked in Cordova, Spain, under the caliphate of the Omijades. He recommended treating only the good diseases, not the bad ones, a measure to prevent bad opinions about doctors. In 60 chapters of his first book, “Surgery”, he reported on bladder and urethral stones, mostly in agreement with his predecessor Celsus [7]. He used clysters before lithotomy and discussed the difficult removal of huge bladder stones in detail. In contrast to Ammonius, he started using a stone caliper and also introduced a ligature around the penis to prevent backward sliding of a urethral stone during the removal procedure. Next, he seemed to be the first physician to crush stones within the urethra by means of an ancestor of the currently used lithotripter. Abulcasis also mentioned stone removal procedures in female patients, which, however, were not performed by him, but by midwives.

As mentioned above, the monks, respected as the ‘intellectuals’ of the fifth to twelfth centuries, were still performing the ‘old’ medical procedures for stone prevention and removal. Under Charles the Great (768–814) the European monasteries were built up as medical centers [8]. The oldest available handbook from this time is the Codex Bambergensis medicinalis 1, which also included urological prescriptions for treatment of urinary stones, as well as enuresis, bladder and kidney pain. Usually only people living in the monastery were treated. However, some monks were so renowned that even high-ranking people, e.g. emperor Heinrich II (973–1024), who suffered from renal stones, were repeatedly treated and even operated on (bladder stone). Hildegard from Bingen (1098–1179), abbess and the first writing female physician, wrote one famous and later subdivided book (Liber simplicis et liber compositae medicinae), which also dealt with urological diseases and treatment availabilities [9]. She already recognized the modern ‘metabolic syndrome’ as one reason for stone disease next to the then typical notion of infection as the reason for stone disease. However, with her, the epoch of ‘monastery medicine’ came to an end, and, finally, with the fourth Lateran Council, the secularization of medicine was induced.

Treatment of bladder stones was popular in the sixteenth to eighteenth centuries, especially because of the traveling Stein Schneider. The prevalence of kidney stone disease did increase during that time and was found throughout all ages and social groups. The reason for that was the change in nutritional habits. After the pest epidemics, nutrition improved in the sixteenth century, with a steady increase in calorie intake [3]. A comparable situation was found after The Thirty Years’ War, during the second half of the seventeenth century and at the beginning of the 18th century. Another very important risk factor explaining the increase in kidney stone prevalence is the increased intake of alcoholic beverages and, especially, that of wine or must, which was a common drink during that time in all social groups, at least in Europe.

During the late seventeenth century Frère Jacques Beaulieu (1651–1714) even treated patients in the royal household of Louis XIV., as well as those in the well-known hospitals “Hotel Dieu” and “Charité” in Paris. Although he was not a monk, he used the title Frère to appear more trustworthy. As most of the stone removal procedures were unsuccessful or were accompanied by severe side effects, mostly resulting in the patient’s death, he fled from the royal household. However, later, he was the first person to use the lateral approach for a perineal lithotomy, which he had invented together with Johann Jakob Rau as “sectio lateralis” [2]. He then performed approximately 5,000 lithotomies, but unfortunately his method was still accompanied by severe morbidity and mortality [10]. As this stone removal procedure was very painful and combined with great mortality, it was only accepted as the final way of treatment, “an act of pure faith”, as Herman Boerhaave (1668–1738), another important figure of medicine during that era, postulated. He published a booklet, “Metaphylactic Advice”, which recommended an increase in fluid intake, a hot bath for vasodilatation, and exercise to induce stone passage [11]. A specific anecdote from this time is a composition by Marin Marais (1656–1728), a French composer, who described the removal of his bladder stone in a musical piece for bass viol [12]. Worth mentioning also is that bladder stone sufferers even applied lithotomy to themselves, for example, Jan de Dost, a Dutchman living in the seventeenth century. Others marked the day of stone removal more than their annual anniversary, or invented devices to ease their pain (e.g. Benjamin Franklin 1706–1790) [13].

At the end of the eighteenth century, determination of stone composition, as well as urinary constituents, became possible. This led to the finding of (increased) excretion of uric acid in urine and of uric acid as a common stone component by Scheele [14]. Later, various other determinants of kidney stone disease were unraveled, with calcium and oxalate being the more prevalent, but also the more rarely found substances like xanthine or cystine [15]. Next, stone analysis gave evidence that most of the bladder stone patients were boys less then 10 years of age. For example, of 1,463 patients with calcium phosphate stones, 950 (∼65%) were below 10 years of age [16]. Surprisingly, however, this progress in ‘stone research’ was soon forgotten, and most of the research done thereafter was focused on clinical pathology instead.

On 13 January 1824, Jean Civiale (1792–1876) first presented a lithotryptic instrument, which enabled him to crush and then remove a bladder stone via the urethra, at the Neckar Hospital in Paris [16]. He then collected the data of patients from all over Europe to show the superiority of his method over the lithotomy procedure, expressing a one in five mortality rate for lithotomy and a significantly lower mortality rate under the usage of the transurethral instrument, in 1826. However, it is worthwhile mentioning that the latter method was only used in patients with smaller stones that were within reach [13].

Historical evidence has shown a significant increase in kidney stones during the past 100 years, with the exception of the two World Wars. In contrast, the incidence of bladder stones decreased, but only in the developed world. However, a fundamental reason, especially for the latter development, cannot be presented. Could it be an interaction of changes in nutrition, vitamin supplementation, fluid intake, or the reduction of urinary tract infections? Nevertheless, bladder stone disease still remains a significant medical problem in the developing world.

Since the early days, people have wanted to treat kidney stone disease by conservative measures. In this respect a variety of plant ingredients were used, which, according to our experiences today, would lead to an increase in urine volume or reduced pain, or had anti-inflammatory components [3]. Hence, today’s (pediatric) nephrologists’ approach to stone prevention instead of repeated—although now easy—stone removal is based on historical grounds. Nevertheless, stone disease differed, and still differs, through geographic, socio-economic and even religious boundaries.

## Differences in epidemiology

### Geographical distribution

The overall probability that an individual will form stones varies in different parts of the world. The risk of developing urolithiasis in adults appears to be higher in the western hemisphere (5–9% in Europe, 12% in Canada, 13–15% in the USA) than in the eastern hemisphere (1–5%), although the highest risks have been reported in some Asian countries such as Saudi Arabia (20.1%) [17, 18]. The incidence of urolithiasis in a given population is dependent on the geographic area, racial distribution, and socio-economic status of the community. Changes in socio-economic conditions over time, and the subsequent changes in dietary habits, have affected not only the incidence but also the site and chemical composition of calculi. Reno-ureteral calculosis featuring mainly calcium oxalate and phosphate is currently more frequent in economically developed countries, whereas vesical calculosis is fairly widespread in Asia, with calculi composed of ammonium urate and calcium oxalate [19]. An epidemiology study by Asper reveals that the occurrence of urolithiasis in the nineteenth century population in Europe was quite similar to that of the twentieth century in Asia [20]. The analogy was demonstrated for age distribution, stone localization, male/female ratio, and stone composition according to the variables defined in Table 1.

**Table 1:** Frequency of urolithiasis according to age, stone location, gender and stone composition in populations of different socio-economic levels (data adapted from Asper [20] and used with permission)

Variable	Socio-economic level	
	Low	High
Frequency in children	High	Low
Bladder stones (%)	>40	<10
Female patients (%)	<20	>25
Calcium oxalate (%)	<40	>60
Uric acid (%)	>30	<20

Stone composition has changed substantially over the past decades, with a progressive increase in frequency of calcium oxalate and calcium phosphate stones, even in the eastern hemisphere, where these stones have been traditionally less frequent than uric acid and infection stones. Recent epidemiology studies from different continents and countries report that calcium oxalate accounts for 60% to 90% of stones in children, followed by calcium phosphate (10–20%), struvite (1–14%), uric acid (5–10%), cystine (1–5%), and mixed or miscellaneous (4%) [21–35].

Hypercalciuria is recognized worldwide as the most frequent underlying factor in calcium oxalate stones, although, in some countries of the eastern hemisphere, hypocitraturia has been reported as the leading cause [36, 37]. Other less frequent metabolic risk factors reported in these studies are hyperuricosuria and hyperoxaluria. However, increased urinary oxalate excretion might be underestimated and might even be a more prevalent risk factor than hypercalciuria for stone disease in some populations (our own experience).

Struvite or infection-related stones, very common in children until the last century, are rarely seen today in industrialized countries, possibly due to improved management of both pediatric obstructive uropathy and urinary tract infections [38]. Nevertheless, epidemiological studies from various countries continue to report a frequency of struvite stones of between 25% and 38% [39–42]. Bladder stones based on malnutrition during the first years of life are currently a frequent finding in various areas of Turkey, Iran, India, China, Indochina and Indonesia [43, 44], although the incidence is proportionally decreasing as social conditions improve. The incidence of bladder stones has been gradually decreasing during the past 100 years in Europe, with a steeper slope in some Asian countries where this tendency has changed quite significantly since the 1980s [21–23, 45–48].

This trend defined, as “stone wave”, has been explained in terms of changing social conditions and the consequent changes in eating habits. In Europe, Northern America, Australia, Japan, and, more recently, Saudi Arabia, affluence has spread to all social classes and with it the tendency for individuals to increase protein intake and to eat rich food in large quantities [19].

**Fig. 1:**
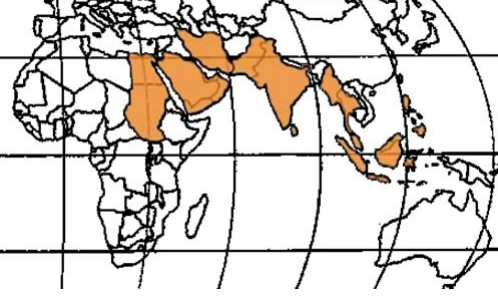
North African–Asian stone belt

The Afro-Asian stone-forming belt stretches from Sudan, the Arab Republic of Egypt, Saudi Arabia, the United Arab Emirates, the Islamic Republic of Iran, Pakistan, India, Myanmar, Thailand, and Indonesia to the Philippines (Fig. 1). In this area of the world, the disease affects all age groups, from less than 1 year old to more than 70 years old, with a male-to-female ratio of 2 to 1. The prevalence of calculi ranges from 4% to 20% [49]. The higher prevalence of urolithiasis in many of those countries is possibly determined by the high consanguinity that prevails among ethnic groups that live in those geographical areas and which may reach 72% according to recent studies [24].

Several studies from northeast Thailand have confirmed the high prevalence of endemic metabolic disorders such as renal tubular acidosis (RTA) as well as a high prevalence of renal stone and hypocitraturia in the same population [50]. Other risk factors involved in this geographical pattern are cultural practices such as the chewing of betel quid, which is common in many countries of the world, particularly in Southeast Asia. The quid consists of a preparation of areca nut, betel leaf and calcium hydroxide ‘lime’ paste, which produces a high incidence of hypercalciuria and hypocitraturia [51]. In the North Indian population the absence of *Oxalobacter formigenes*, an intestinal oxalate degrading bacteria, can lead to a significant increase in the risk of absorptive hyperoxaluria, resulting in recurrent episodes of calcium oxalate stones [52]. Although most of the adult population has been found to be intestinally colonized by *Oxalobacter* in other regions of the world, like the Ukraine or Florida, USA [53], a recent paper by Kaufman et al. has suggested that the absence of *Oxalobacter* is one significant risk factor in patients with kidney stones [54].

**Fig. 2:**
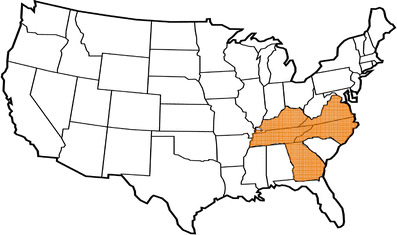
North American stone belt

In the USA, nephrolithiasis is said to be responsible for 1 in 7,600 to 1 in 1,000 pediatric hospital admissions [25, 55]. A recent study, however, has reported a fivefold increase in the incidence of pediatric urolithiasis during the past decade [26]. The incidence varies by region, but urolithiasis is most common in the southeastern region of the country, where the states of Virginia, North Carolina, Georgia, Tennessee, and Kentucky are described collectively as the North American “stone belt” [27] (Fig. 2).

Soucie et al. have recently published a nationwide study that included information on self-reported, physician-diagnosed, kidney stones collected from 1,167,009 men and women from all 50 US states. After adjustment for age and race, the odds of ever having had kidney stones increased from west to east and from north to south. The odds of stones among participants who resided in the southeast were nearly twice that of those living in the northwest [56]. North American studies report that 75% of children who have urolithiasis have an identifiable predisposition to stone formation and metabolic risk factors such as hypercalciuria and hyperuricosuria that account for more than 50% of cases [57].

There are few pediatric epidemiologic studies from other countries of the American continent. A study from Venezuela reported that urolithiasis was responsible for 7% of general outpatient consultations in all national children’s hospitals during 1998 [28]. In Chile, the reported rate of pediatric urolithiasis was 1.6 in 1,000 pediatric admissions and 4.3% of pediatric nephrology admissions during 2003 [29].

In summary, the epidemiology of renal stones with regard to stone composition is continuing to change all over the world towards a predominance of calcium oxalate stones. Major differences in the frequency of the other constituents, particularly uric acid and struvite, reflect particular eating habits and infection risk factors specific to certain population.

### Race and gender

Idiopathic stone disease occurs more frequently in white Caucasians than in Blacks, irrespective of the geographic area concerned. In the USA and Brazil, the same 4 to 1 Caucasian-to-Black ratio between stone formers was reported [58]. Probably, these differences cannot simply be accounted for by inborn racial factors. Indeed, there was a significant increase in the prevalence of urolithiasis in Black Americans once they had adopted Caucasian dietary habits [58]. With regard to gender distribution, the male-to-female ratio appears to be higher in White populations than in Black Americans and Hispanics [58–61].

### Inheritance and familial recurrence

Autosomal recessive inheritance was defined for cystinuria and primary hyperoxaluria. The reported prevalence for cystinuria is 1–5% of all patients with urolithiasis and much lower for primary hyperoxaluria (∼2 per million populations). These diagnoses require sophisticated procedures, which make epidemiological surveys rather rare in underdeveloped countries [62]. Prevalence rates for primary hyperoxaluria are higher in regions with a high rate of consanguineous marriages, such as in Northern Africa or in Israeli Arabs [63, 64]. Cystinuria and primary hyperoxaluria, as well as renal tubular acidosis and Dent’s disease, are some of the different monogenic conditions that have been identified to date as etiologies for urolithiasis. However, all of these rare conditions probably account for less than 2% of renal stones [65]. A familial occurrence has also been suggested for hypercalciuria, one of the main risk factors for idiopathic urolithiasis [66–69]. However, familial recurrence does not necessarily imply an inherited transmission, as it may be an effect of environmental factors shared by family members, mainly those related to dietary habits. A study by Curhan et al. suggests that approximately 60% of the enhanced risk of stone formation among relatives of patients with idiopathic urolithiasis might be related to genetic inheritance [70]. Similar results were reported by Goldfarb and colleagues in a large twin study to examine genetic and non-genetic factors associated with stones [71]. To date, the available data suggest that, in the vast majority of kidney stone formers, the mode of inheritance is complex and polygenic.

### Climate and season

It has been well documented that the incidence of urinary stones is higher in countries with warm or hot climates, probably due to low urinary output and scant fluid intake. These are some of the factors that contribute to the geographical pattern that has characterized the North American and Afro-Asian stone belts. Also, in a given population, stone recurrence is higher in summer and fall than in winter and spring [48]. In a North American study the prevalence of stones tended to increase as the average annual temperature (5.2°C in North Dakota to 22°C in Florida) and sunlight index (14.6 in Washington state to 39.7 in Florida) increased [56].

### Dietary habits

Epidemiologic observations leave no doubt that diet plays a major, if not the most important, role in the pathogenesis of urolithiasis. Much evidence has been put forward that the consumption of animal protein is closely related to the prevalence of stone disease in a given population. Animal protein intake has a great influence on the whole stone forming risk and the chemical composition of urinary calculi [72–74].

Recent studies report that actual protein consumption in children in Europe and North America is three-time to five-times higher than recommended [75]. A meta-analysis of the data from a variety of studies in children has been used to derive values for the average protein requirements and for a safe level of protein intake. Protein requirements range from 1.12 g/kg per day at age 6 months to 0.74 g/kg per day at 10 years, followed by a small decline towards the adult value in adolescence. Safe values of protein intake are said to range from 1.43 g/kg per day at 6 months to 0.91 g/kg per day at 10 years [76].

The higher prevalence of urolithiasis in Saudi Arabia than in the USA and Europe has been ascribed to a high intake of animal protein, which was 10% and 50% higher than in the USA and Europe, respectively [18]. The prevalence of uric acid and calcium oxalate stones also appeared to be influenced by animal protein in the diet. In Japan, bladder and upper urinary tract stones shared the same prevalence in 1950, but the latter had risen to 95% by 1980. During the same interval, individual animal protein intake in Japan rose from 20 g per day to 40 g per day [45].

This increase in upper urinary tract stone prevalence also occurred in other eastern countries where consumption of animal and vegetable proteins had increased during the past decades [48].

A number of mechanisms have been proposed to explain the metabolic changes induced with increased protein consumption [77]: (1) protein contains amino acids composed of sulfur, such as cystine and methionine, which are more prevalent in animal protein. Sulfur is oxidized, yielding sulfate, which generates an acid load that is buffered by bone. The resultant osseous dissolution provides more calcium to be excreted [78–81]. (2) Sulfate also forms a soluble complex with calcium in the nephron and limits the reabsorption of this cation. (3) Increased protein consumption augments glomerular filtration, thus delivering more calcium to the nephron [82–86]. (4) Animal protein has a high purine content, which explains the associated increase in uric acid excretion. This is a risk factor for the development of uric acid stones and may play a role in calcium stone formation. (5) Chronic metabolic acidosis induced by the increased acid load decreases calcium reabsorption within the nephron [85–87]. (6) The decreased urinary pH may potentiate uric acid lithiasis, and it enhances citrate reabsorption in the proximal tubules, thus decreasing the excretion of this important inhibitor of crystallization [88]. (7) The augmented oxalate excretion with increasing dietary protein reported by some investigators may be caused by generation of more glycolate, an oxalate precursor [89].

As well as a protein-rich diet that enhances the risk for calcium oxalate and upper urinary tract stones, a nutritionally poor diet that is low in animal protein, calcium and phosphate but high in cereal and therefore acidogenic is the main factor that leads to the development of bladder stones in children in underdeveloped countries. This leads to the formation of urine with a relatively high content of ammonium and urate ions and, consequently, to the formation of ammonium acid urate stones [21].

Sodium intake has a major role as a risk factor for stone formation in view of the fact that high urinary sodium excretion has been repeatedly associated with hypercalciuria in adult and pediatric populations [90, 91]. Sakhaee et al. have shown that sodium loading in healthy adult subjects increases urinary pH and the relative supersaturation ratios of brushite and monosodium urate but reduces urinary citrate excretion and serum bicarbonate levels [92]. The increased calcium excretion is thought to be caused by sodium-induced expansion of the volume of extracellular fluid, resulting in inhibition of calcium reabsorption in the nephron. The decreased citrate excretion is ascribed to the effect that bicarbonate has on citrate metabolism in proximal tubular cells [93]. The increase in urinary pH is likely caused by the increased bicarbonate excretion.

An inverse relationship occurs between renal potassium and calcium excretion, which brings attention to the role of potassium-rich foods such as vegetables and fruits in the prevention of stone formation [94, 95]. Potassium consumption augments renal tubular phosphate absorption, which inhibits the synthesis of 1,25-dihydroxyvitamin [96, 97]. This results in decreased intestinal absorption of calcium, which reduces urinary calcium excretion. Another potential benefit is that foods high in potassium content are usually replete with alkali, which reflects the dietary intake of actual bicarbonate or potential bicarbonate that reduces net acid excretion and stimulates urinary citrate excretion [98].

The importance of dairy products in the pathogenesis and treatment of urolithiasis has been debated for decades. Dairy products play a twofold pivotal role, being a source of both animal protein and calcium. Curhan et al. assessed the relative role of calcium intake as a dietary risk factor for urolithiasis among 40,000 healthy subjects in the USA. Their study demonstrated an increasing risk for developing urolithiasis with decreasing calcium intake from over 1,050 mg/day to 600 mg/day [99]. These findings were explained by the well-known effect of dietary calcium on intestinal absorption of oxalate. The preceding arguments and the definite importance of an adequate calcium intake for normal bone metabolism in childhood are the basis for avoiding restrictive dietary prescriptions that are below the calcium requirements for different age groups.

There is evidence that carbohydrate ingestion has an impact on certain recognized stone risk factors. Carbohydrate consumption induces increased urinary calcium excretion, which has been ascribed to decreased distal tubular calcium absorption and augmented intestinal calcium uptake [78, 100–102]. Several investigations have demonstrated that urinary oxalate excretion significantly increases after administration of a glucose load [103]. Recent studies have suggested an increased prevalence of urolithiasis and recurrence associated with obesity, with elevated urinary excretion of calcium, sodium, uric acid and oxalate [104–107]. In obese patients, associated metabolic derangements such as insulin resistance and compensatory hyperinsulinemia may lead to the formation of calcium-containing kidney stones [108]. A recent metabolic trial demonstrated that insulin resistance was associated with defects in renal ammonium ion production [109]. A defect in renal acid excretion could lead to hypocitraturia, an important risk factor for calcium urolithiasis. Hyperinsulinemia may also contribute to the development of calcium stones by increasing the urinary excretion of calcium [110].

It appears that the more we deviate from a traditional balanced diet, with adequate intake of protein, calcium, salt, fruits, and vegetables, and supplement it with rich fast foods and artificial drinks, the more we increase our risk for urolithiasis. Therefore, a diet that contains recommended daily allowances of protein and calcium and is low in sodium and oxalate and rich in potassium is recommended, not only for children with hypercalciuria, hyperuricosuria, oxaluria, and idiopathic stones, but also for the prevention of stone formation in the pediatric and adult population.

## Conclusion

Both historical and current epidemiological data show a high variety of pathophysiological data for recurrent stone disease. However, one observation is true for every individual region of the world: the incidence of stone disease in children and adolescents is increasing, as it is in adults. Hence, knowledge of regional diversities is necessary if we are to perform a proper examination of the patient with a kidney stone. The available evidence with regard to the importance of dietary habits in the pathogenesis of kidney stone formation brings forward the major role that general pediatricians and pediatric nephrologists play in the guidance of their patients’ families with respect to adequate nutrition and fluid intake. This kind of intervention is surely the less costly and less painful for one of the most preventable renal conditions and was already the most popular recommendation centuries ago!

## Questions

(Answers appear following the reference list)

### History

1. Historical evidence of urinary calculi has been found from as long ago as:
  - 500 years BC
  - 1,500 years BC
  - 5,000 years BC
  - 8,000 years BC
2. The procedure of lithotomy was first mentioned
  - 3,000 years BC
  - 2,000 years BC
  - 1,000 years BC
  - 300 years BC
3. The first report on stone removal via the urethra came from
  - Egypt
  - India
  - Arabia
  - Europe
4. The reasons for the prohibition by the Hippocratic Oath of lithotripsy procedure in the second and third centuries BC were the risks of complications such as:
  - High fevers
  - Urinary fistulas
  - Impotence
  - Death
5. The increase in prevalence of kidney stone disease during the sixteenth to eighteenth centuries, specially in Europe, was due to:
  - Changes in dietary habits
  - Increase in calorie intake
  - Increased intake of alcoholic beverages
  - All of the above

### Epidemiology

6. The higher incidence of urolithiasis in countries of the so-called North African–Asian stone belt is possibly determined by:
  - Warm climate
  - Low protein intake
  - High consanguinity
  - High intake of cereals
7. The location of urinary calculi in economically developed countries is most frequent in the:
  - Upper urinary tract
  - Bladder
  - Urethra
  - Could be in either one
8. The trend defined as “stone wave” has been explained in terms of:
  - Changing social conditions
  - Increase in protein intake
  - Increase in “rich” food intake
  - All of the above
9. In the United States of America an identifiable predisposition to stone formation may be demonstrated in:
  - Fewer than 10% of patients with nephrolithiasis
  - 20–50% of patients with nephrolithiasis
  - 75% patients with nephrolithiasis
  - 80–90% patients with nephrolithiasis
10. Dietary factors that are related to high incidence of nephrolithiasis are:
  - High protein intake
  - High sodium intake
  - Low potassium intake
  - All of the above
